# Developing and validating a risk algorithm to diagnose *Neisseria gonorrhoeae* and *Chlamydia trachomatis* in symptomatic Rwandan women

**DOI:** 10.1186/s12879-021-06073-z

**Published:** 2021-04-28

**Authors:** Kristin M. Wall, Julien Nyombayire, Rachel Parker, Rosine Ingabire, Jean Bizimana, Jeannine Mukamuyango, Amelia Mazzei, Matt A. Price, Marie Aimee Unyuzimana, Amanda Tichacek, Susan Allen, Etienne Karita

**Affiliations:** 1 Rwanda Zambia HIV Research Group, Department of Pathology & Laboratory Medicine, School of Medicine and Hubert Department of Global Health and Department of Epidemiology, Rollins School of Public Health, Laney Graduate School, Emory University, 1518 Clifton Road NE, Atlanta, GA 30322 USA; 2 Projet San Francisco, Rwanda Zambia HIV Research Group, Kigali, Rwanda; 3 IAVI, NY, NY, University of California San Francisco, San Francisco, CA 94115 USA

**Keywords:** *Neisseria gonorrhoeae*, *Chlamydia trachomatis*, Rwanda, Risk algorithm

## Abstract

**Background:**

Algorithms that bridge the gap between syndromic sexually transmitted infection (STI) management and treatment based in realistic diagnostic options and local epidemiology are urgently needed across Africa. Our objective was to develop and validate a risk algorithm for *Neisseria gonorrhoeae* (NG) and *Chlamydia trachomatis* (CT) diagnosis among symptomatic Rwandan women and to compare risk algorithm performance to the current Rwandan National Criteria for NG/CT diagnosis.

**Methods:**

The risk algorithm was derived in a cohort (*n* = 468) comprised of symptomatic women in Kigali who sought free screening and treatment for sexually transmitted infections and vaginal dysbioses at our research site. We used logistic regression to derive a risk algorithm for prediction of NG/CT infection. Ten-fold cross-validation internally validated the risk algorithm. We applied the risk algorithm to an external validation cohort also comprised of symptomatic Rwandan women (*n* = 305). Measures of calibration, discrimination, and screening performance of our risk algorithm compared to the current Rwandan National Criteria are presented.

**Results:**

The prevalence of NG/CT in the derivation cohort was 34.6%. The risk algorithm included: age < =25, having no/primary education, not having full-time employment, using condoms only sometimes, not reporting genital itching, testing negative for vaginal candida, and testing positive for bacterial vaginosis. The model was well calibrated (Hosmer-Lemeshow *p* = 0.831). Higher risk scores were significantly associated with increased prevalence of NG/CT infection (*p* < 0.001). Using a cut-point score of > = 5, the risk algorithm had a sensitivity of 81%, specificity of 54%, positive predictive value (PPV) of 48%, and negative predictive value (NPV) of 85%. Internal and external validation showed similar predictive ability of the risk algorithm, which outperformed the Rwandan National Criteria. Applying the Rwandan National Criteria cutoff of > = 2 (the current cutoff) to our derivation cohort had a sensitivity of 26%, specificity of 89%, PPV of 55%, and NPV of 69%.

**Conclusions:**

These data support use of a locally relevant, evidence-based risk algorithm to significantly reduce the number of untreated NG/CT cases in symptomatic Rwandan women. The risk algorithm could be a cost-effective way to target treatment to those at highest NG/CT risk. The algorithm could also aid in sexually transmitted infection risk and prevention communication between providers and clients.

**Supplementary Information:**

The online version contains supplementary material available at 10.1186/s12879-021-06073-z.

## Introduction

The World Health Organization (WHO) estimates that *Neisseria gonorrhoeae* (NG) and *Chlamydia trachomatis* (CT) prevalence has increased more than other curable sexually transmitted infections (STIs) in Africa [1]. Infection with NG or CT has been shown to increase risk of HIV transmission [2–5] and, in women, to increase risk of pelvic inflammatory disease, infertility, and preterm birth [6].

Syndromic STI management systems [7–14] remain the standard of care across much of Africa due to the high cost of culture-based and molecular diagnostics. While STI symptoms may be useful in identifying STIs in men, they are less easily interpreted in women who often experience non-STI genital conditions that produce discharge [15]. A particular challenge with syndromic management of women is distinguishing causes of endocervical infection (NG/CT) from vaginal infection or dysbiosis (*Trichomonas vaginalis* (TV), bacterial vaginosis (BV), and vaginal *Candida albicans* (VCA)).

Per the 2019 Rwandan National Criteria [16], management of men presenting with urethral discharge is presumptive treatment for NG/CT. Management of women presenting with vaginal discharge requires report of at least two of the following three risk factors to be treated for NG/CT cervicitis: age < 21, single, and > =2 sexual partners. Though WHO calls for development of locally relevant algorithms to improve STI diagnosis [17], the 2019 Rwanda criteria for women are not based on the local NG/CT prevalence or correlates of NG/CT.

We recruited symptomatic men and women in Kigali between January 2016 and August 2019 using radio announcements and referrals for free point-of-care STI screening and treatment at the Center for Family Health Research (CFHR) site in Kigali, Rwanda. We found very high NG and CT prevalence in men (among 1013 tests, 74% were NG positive, 20% CT positive, and 19% were negative) (Wall et al., under review). These data support that syndromic treatment of NG/CT in men according to the 2019 Rwandan National Criteria [16] may perform relatively well. In contrast, while we also found high NG and CT prevalence in women, the clinical picture was more complex (among 579 tests, 26% were NG positive, 17% CT positive, 21% BV positive, 21% VCA positive, 13% TV positive, 30% negative) (Wall et al., under review).

Here, our objective is to develop and validate a risk algorithm for diagnosis of NG/CT among symptomatic women in Rwanda and to compare the performance of this algorithm with the 2019 Rwandan National Criteria [16].

## Methods

### Ethics

This program was approved as non-research by the Rwandan National Ethics Committee and the US Centers for Disease Control (CDC) that funded service provision through PEPFAR. This program also met non-research criteria of the Emory Institutional Review Board. STI diagnosis and treatment were provided anonymously as free public health services. All methods were carried out in accordance with relevant guidelines and regulations.

### Derivation cohort

Women were recruited between January 2016 and August 2019 in Kigali, the capital of Rwanda. Radio announcements encouraged men and women with genital discharge, discomfort, or ulcer to seek free STI screening and treatment services at our CFHR site from trained staff. Clients were asked to refer sex partners and any known symptomatic person. Pharmacists were asked to refer people seeking treatment for STI-suggestive symptoms to the CFHR site. We limited the derivation cohort to symptomatic women, defined as reporting vaginal discharge, to be comparable with the Rwandan National criteria. Some women returned for additional care, confirmation of treatment effectiveness, or other reasons. The final risk algorithm derivation cohort was composed of women’s first visits only.

### External validation cohort

Between January to March 2020, symptomatic women were again recruited for free STI screening and treatment at our CFHR site using the same recruitment strategies described above. However instead of trained site staff, STI diagnoses were made by eight government clinic providers who were trained by our staff during a didactic training session (with pre/post-training assessment) on STI etiologies, treatments, and use of the Rwandan National Criteria for diagnosis among symptomatic patients. After government providers made diagnoses, patients underwent gold standard STI diagnosis and treatment as described below. The final external validation cohort was composed of women’s first visits only.

### Survey, genital exam, and laboratory measures

In both the derivation and external validation cohort, demographic, risk factor, and symptom data were collected via standardized surveys. Survey questions were identified through literature review and based on our previous work describing STIs in Rwanda [18–21]. Surveys were administered by nurses who entered participant responses into MS Access. Genital exams were performed by trained physicians and nurses. We conducted laboratory testing of patient samples including phlebotomy for rapid HIV and rapid plasma reagin (RPR) serologies; microscopy of vaginal swab wet mount preparations to diagnose TV, BV and VCA; and endocervical swabs for GeneXpert (Cepheid, Sunnyvale USA) testing for NG and CT. Given concerns about emergence of antibiotic resistance of NG in Rwanda and elsewhere in Africa [12, 22] and the dated information from Rwanda (last published study in 2000) [23] we requested patients treated for NG return for retesting 2–3 weeks after treatment. National guidelines (2015) for first-line NG treatment specified ciprofloxacin with ceftriaxone used in cases of resistance, and doxycycline for CT. This changed in the 2019 Guidelines to ceftriaxone as first-line treatment for NG.

### Data analyses

Analyses are conducted with Statistical Analysis Software version 9.4 (Cary, NC).

#### Outcome of interest

Our outcome of interest was NG and/or CT infection diagnosed on GeneXpert in women presenting with vaginal discharge. We recognize and discuss below the difference between a positive nucleic acid test and a confirmed infection. We focus on symptomatic women and combined NG and CT to be comparable with the Rwandan National Criteria for diagnosis of NG/CT.

#### Baseline characteristics and associations with NG or CT infection

Demographic, behavioral, and symptom data are described for the derivation and validation cohorts. Counts and percentages (categorical variables) and means and standard deviations (continuous variables) are presented. The data were described overall and by NG/CT infection status. Chi-square (or Fisher’s exact) or t-tests evaluated whether differences in the distribution of baseline data by NG/CT infection status was due to chance.

#### Derivation and calibration of the risk algorithm

Bivariate and multivariable logistic regression models identified variables associated with the outcome of interest. Variables were included in multivariable models if they were associated (*p* < 0.05) with the outcome in bivariate analyses, survived backward elimination, and were considered feasible measures in a Rwandan government clinic setting (for example, phlebotomy and microscopy are available in most health centers while physical exams are not). Variable multi-collinearity was assessed. Crude and adjusted prevalence odds ratios, 95% confidence intervals, and *p*-values were calculated. Score values for individual variables in the final model were obtained by dividing each variable’s estimated model coefficient by the lowest coefficient among all variables and rounding to the nearest integer. To assess model calibration, we used the Hosmer-Lemeshow Goodness-of-Fit test using the LACKFIT option in SAS.

#### Internal validation of the risk algorithm

We used standard 10-fold cross validation methods [24] for internal validation. Briefly, variables significantly (*p* < 0.05) associated with the outcome in bivariate analysis were included in initial multivariate models excluding a random 1/10th of the data. The final model was derived by backwards elimination and a model coefficient-weighted score was created from the variables retained in the final model. The scores derived were then tested in the remaining 1/10th of the data. This process was repeated ten times such that each 1/10th was withheld and then tested in turn. Then the same process was applied to ten 90% training sets and 10% test sets.

#### Discrimination of the risk algorithm and the Rwandan National Criteria

The area under the receiver operating curve (AUC) was calculated using standard methods [25] for the risk algorithm applied to the derivation cohort, after 10-fold cross validation (average AUC of the 10 different models is presented), and applied to the external validation cohort. The AUC was also calculated after applying the Rwandan National Criteria to the derivation cohort. Receiver operating curves were graphed and compared for the risk algorithm versus the Rwandan National Criteria, both as applied to the derivation cohort. Measures of sensitivity, specificity, positive predictive value (PPV), and negative predictive value (NPV) of the risk algorithm were calculated using score cut-offs defined as the median score.

#### Distribution of NG/CT prevalence and population by score categories

The prevalence of NG/CT within risk score categories were calculated in the derivation cohort comparing the risk algorithm with the Rwandan National Criteria. The population distribution of the derivation cohort falling within each risk score category was also calculated.

## Results

### Baseline characteristics and associations with NG or CT infection

Most (82%) women were symptomatic. Among the *n* = 468 symptomatic women in the derivation cohort, the prevalence of NG/CT infection was 35% (Table 1). Most women reported only one or no partner in the last month (83%) and most (64%) reported never using condoms during vaginal sex in the last 3 months. Roughly one-fifth of women were BV positive and one-fifth were candida positive. Roughly half had endocervical inflammation or discharge on physical exam. Overall, the derivation and external validation cohorts were similar. Among the *n* = 305 women in the external validation cohort, the prevalence of NG/CT was 28% (Supplemental Table 1). Some differences include the prevalence of BV (21% derivation versus 40% validation cohort), and endocervical inflammation or discharge on physical exam (53% derivation versus 36% validation cohort).

**Table 1:** Baseline characteristics and associations with NG or CT infection in symptomatic women, Kigali (*N* = 468): derivation cohort

	Total (***N*** = 468)		Either NG or CT (***n*** = 162)		NG and CT Uninfected (***n*** = 306)		***p***-value
	N	Col %	N	Row %	N	Row%	
**Demographics**							
**Age**							
25 or younger	187	40%	86	46%	101	54%	< 0.0001
Older than 25	281	60%	76	27%	205	73%	
**Referrer**							
Radio Advert	233	50%	70	30%	163	70%	0.038
Other^a^	235	50%	92	39%	143	61%	
**Living and Marital Status Composite**							
Married and Cohabiting	216	46%	57	26%	159	74%	0.001
Other	252	54%	105	42%	147	58%	
**Education Level**							
None/Primary	256	55%	75	29%	181	71%	0.008
Secondary/Higher	212	45%	87	41%	125	59%	
**Employment Status**							
Full-time employment	165	35%	43	26%	122	74%	0.004
Part-time/Student/Jobless	302	65%	119	39%	183	61%	
**Sexual behaviors**							
**Number of partners in last 30 days**							
None or one partner	355	83%	102	29%	253	71%	< 0.0001
More than one partner	72	17%	44	61%	28	39%	
**Condom use during vaginal sex in the last three months**							
Always (or did not have vaginal sex)	30	7%	7	23%	23	77%	< 0.0001
Sometimes	123	29%	68	55%	55	45%	
Never	274	64%	71	26%	203	74%	
**Number of days since sexual contact you suspect STI was acquired from**							
0–16	90	21%	42	47%	48	53%	0.006
>=17	339	79%	106	31%	233	69%	
**Self-reported symptoms**							
**Genital itching**							
Yes	266	57%	72	27%	194	73%	<.001
No	199	43%	87	44%	112	56%	
**Number of days with symptoms**							
1–10	113	21%	48	42%	65	58%	0.029
11 or more	321	60%	100	31%	221	69%	
**HIV and Other STI Results**							
**HIV Status**							
Positive	59	13%	29	49%	30	51%	0.012
Negative	409	87%	133	33%	276	67%	
**RPR Result**							
Positive (1, 11 or greater)	39	8%	23	59%	16	41%	0.001
Negative	423	92%	138	33%	285	67%	
**Candida**							
Positive	106	23%	17	16%	89	84%	< 0.0001
Negative	348	77%	138	40%	210	60%	
**BV**							
Positive	96	21%	49	51%	47	49%	< 0.0001
Negative	355	79%	104	29%	251	71%	
**Physical exam**							
**Vaginal Inflammation or Discharge**							
Yes	398	92%	129	32%	269	68%	0.014
No	36	8%	19	53%	17	47%	
**Endocervical Inflammation or Discharge**							
Yes	230	53%	96	42%	134	58%	0.001
No	202	47%	52	26%	150	74%	

^a^Heard from Friends/Walk-in/Pharmacy/Other/Invitation/Contact Partner/Internet

Not associated, not tabled: number of children under 18, number of additional children desired, pregnancy status, wanting more children in the next two years, family planning method, burning sensations when passing urine, genital ulcer, dyspareunia, unpleasant odor, lower abdominal pain, trichomonas, genital ulcer

*CT* Chlamydia trachomatis, *NG *Neisseria gonorrhoeae, *SD* standard deviation, *STI* sexually transmitted infection, *RPR* rapid plasma reagin, *BV* bacterial vaginosis

### Risk algorithm components, calibration, and discrimination

As shown in Table 2, the risk algorithm included being 25 or younger (2 points), having no/primary education (1 point), not having full-time employment (1 point), sometimes using condoms (2 points), not reporting genital itching (2 points), not having candida (2 points), and having BV (1 point). The Hosmer-Lemeshow Goodness-of-Fit test indicated good calibration (*p*-value = 0.831). The risk algorithm had reasonable discrimination in the derivation cohort (AUC = 0.75, 95%CI: 0.70–0.79, *p* < 0.001). We also had reasonable discrimination in the 10-fold cross validation (AUC = 0.71, 95%CI: 0.55–0.86, *p* < 0.01, Table 3) and the external validation cohort (AUC = 0.63, 95%CI: 0.56–0.70, p < 0.01).

**Table 2:** Risk score algorithm comprised of factors associated with NG or CT infection in symptomatic women, Kigali (*N* = 468)

	Final multivariable model				Score points
	aPOR	95% CI		***p***-value	
**Age**					
25 or younger	2.38	1.47	3.84	<.001	2
Older than 25	ref				
**Education Level**					
None/Primary	1.68	1.04	2.72	0.034	1
Secondary/Higher	ref				
**Employment Status**					
Full-time employment	ref				
Part-time/Student/Jobless	1.76	1.05	2.94	0.032	1
**Condom use during vaginal sex in the last three months**					
Always (or did not have vaginal sex)	1.06	0.40	2.84	0.906	
Sometimes	2.51	1.52	4.14	<.001	2
Never	ref				
**Genital itching**					
Yes	ref				
No	2.28	1.43	3.66	0.001	2
**Candida**					
Positive	ref				
Negative	2.76	1.46	5.22	0.002	2
**BV**					
Positive	1.99	1.17	3.38	0.011	1
Negative	ref				

*CT* Chlamydia trachomatis, *NG* Neisseria gonorrhoeae, *aPOR* adjusted prevalence odds ratio, *CI* confidence interval, *BV* bacterial vaginosis

Area Under the Curve: 0.75 (95%CI: 0.70–0.79, p < 0.001)

Hosmer-Lemeshow: Chi-square = 4.29, *p* = 0.831

**Table 3:** Results of 10-fold cross validation or a risk score algorithm comprised of factors associated with NG or CT infection in symptomatic women, Kigali (*N* = 468)

Test Group	AUC	95%CI		***p***-value
10	0.71	0.54	0.89	0.019
9	0.69	0.51	0.86	0.037
8	0.83	0.70	0.96	< 0.0001
7	0.66	0.50	0.81	0.048
6	0.72	0.57	0.86	0.003
5	0.68	0.51	0.84	0.039
4	0.73	0.57	0.89	0.005
3	0.76	0.63	0.90	< 0.001
2	0.72	0.58	0.86	0.003
1	0.60	0.43	0.76	0.258
**Average**	0.71	0.55	0.86	< 0.01

*CT* Chlamydia trachomatis, *NG *Neisseria gonorrhoeae, *AUC* area under the curve, *CI* confidence interval

### Discrimination of the risk algorithm compared to the Rwandan National Guideline Criteria

**Fig. 1:**
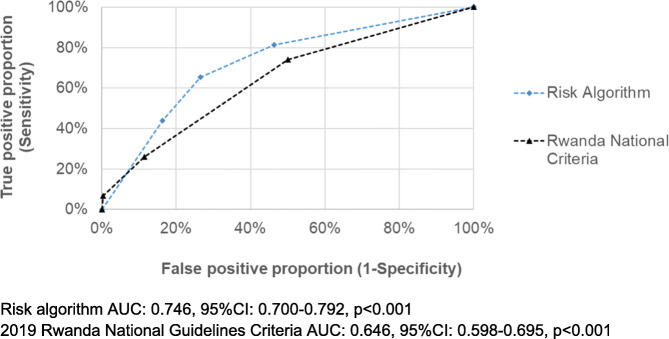
Receiver operating curves companing the risk algorithm and Rwandan National Criteria (derivation cohort, *n* = 468 women)

Sensitivity, specificity, PPV, and NPV for the risk algorithm and the Rwandan National Criteria are shown in Table 4. The risk algorithm in the derivation cohort had 81% sensitivity, 54% specificity, 48% PPV, and 85% NPV for a score cutoff of > = 5. As shown in Table 4, the risk algorithm performed similarly in the external validation cohort. In comparison, the Rwandan National Criteria was substantially less sensitive than the risk algorithm. Applying the Rwandan National Criteria cutoff of > = 2 (the cutoff level in 2019 Guidelines) to our derivation cohort had a sensitivity of 26%, specificity of 89%, PPV of 55%, and NPV of 69%. These findings are reflected in the receiver operating curve shown in Fig. 1. The Rwandan National Criteria had lower discrimination compared to the risk algorithm.

**Table 4:** Risk algorithm performance to identify NG or CT infection in symptomatic women compared with Rwandan National Criteria

	Sensitivity	Specificity	PPV	NPV
**Risk Score**				
**Derivation cohort**				
Score > =4	91%	36%	43%	88%
Score > =5	81%	54%	48%	85%
Score > =6	65%	74%	57%	80%
Score > =7	44%	84%	59%	74%
**External validation cohort**				
Score > =4	79%	33%	32%	80%
Score > =5	67%	48%	34%	79%
Score > =6	53%	66%	38%	78%
Score > =7	35%	79%	39%	76%
**Rwanda National Criteria**				
**Derivation cohort**				
Score > =2^a^	26%	89%	55%	69%

*CT* Chlamydia trachomatis, *NG *Neisseria gonorrhoeae, *PPV* positive predictive value, *NPV* negative predictive value

^a^Current cutoff used in the 2019 Rwandan National Guidelines

### Distribution of NG/CT prevalence and population by score category comparing the risk algorithm to the Rwandan National Criteria

**Fig. 2:**
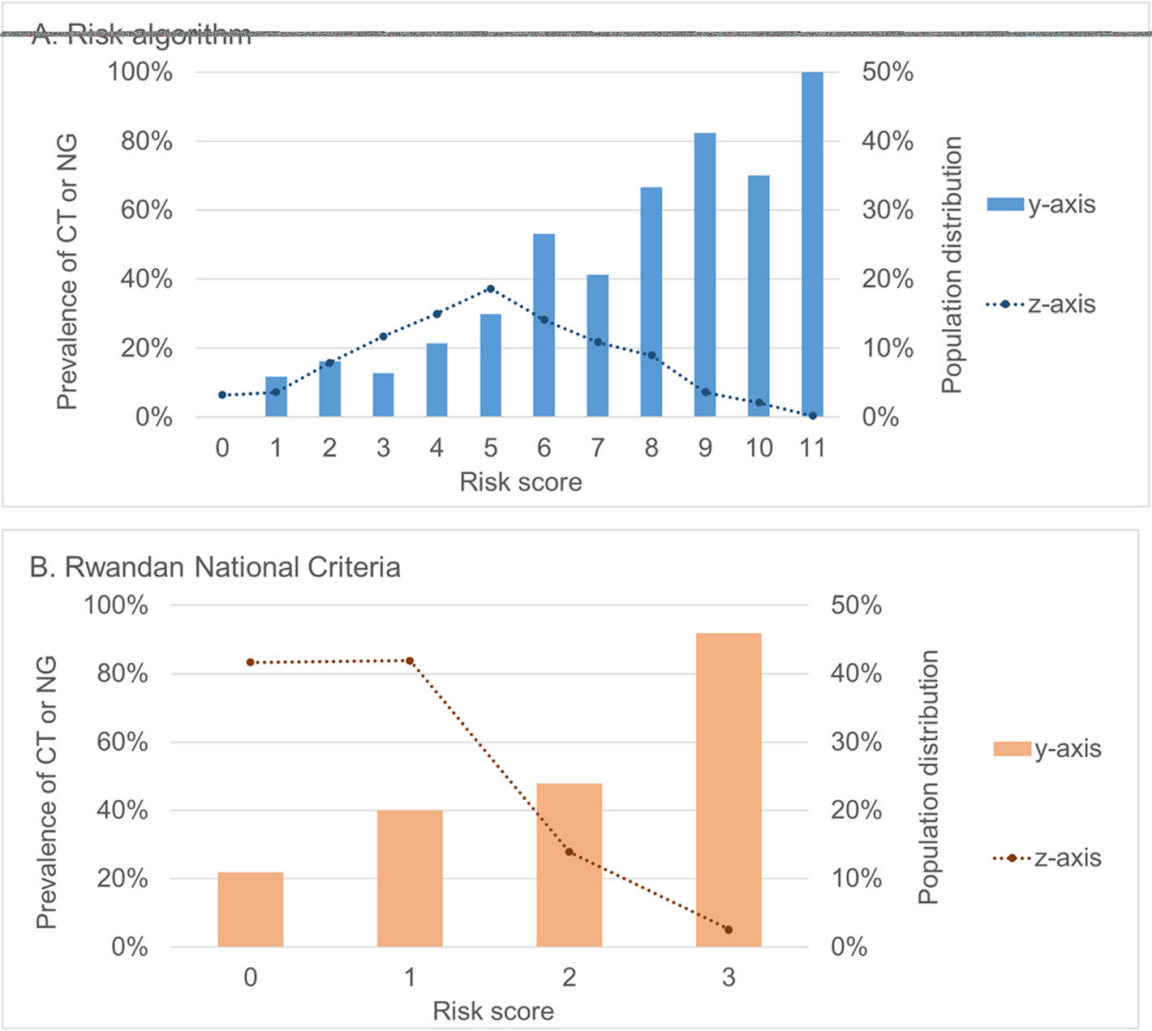
Prevalence of CT/NG and population distribution within exact risk score categories comparing the risk algorithm (panel **a**) and the 2019 Rwandan National Guidelines Criteria (panel **b**) applied to the derivation cohort (*n* = 468 women)

For both the risk algorithm and the Rwandan National Criteria, higher scores were significantly associated with increased prevalence of NG/CT infection (*p* < 0.001) (Fig. 2a and b). Applying the risk algorithm (Fig. 2a), most of the population had risk algorithm scores of 4 (15% of the population), 5 (19%), or 6 (14%). The prevalence of NG/CT within these risk algorithm categories were 21, 30, and 53%, respectively. Applying the Rwandan National Criteria (Fig. 2b), most of the population had risk scores of 0 (42% of the population) or 1 (42%). The prevalence of NG/CT within these risk algorithm categories was 20 and 40%, respectively.

## Discussion

In this study, we developed and validated a simple risk algorithm comprised of demographic, symptoms, and laboratory data and based on up-to-date epidemiological data from Kigali, Rwanda. This risk algorithm outperformed the current Rwandan National Criteria. Importantly, the current Rwandan National Criteria cutoff score of > = 2 would have missed identifying many women who were NG/CT positive in our derivation cohort – the proportion of NG/CT cases *not* diagnosed (i.e., the proportion of false negatives) was 19% using the risk algorithm (cutoff > = 5) compared to 74% using Rwandan National Criteria (cutoff > = 2). These findings exemplify the WHO recommendation that locally relevant data should inform approaches to diagnosis and treatment [17].

The advent of GeneXpert diagnostics for NG and CT has greatly enhanced STI management. A recent study in Rwanda found that providing GeneXpert tests for NG/CT as well as point-of-care tests for BV and TV in symptomatic, high-risk women significantly reduced overtreatment [26]. However, at $18/test for GeneXpert reagents and reliance on an expensive machine that is not widely available, this technology remains unaffordable in much of Africa. Algorithms that bridge the gap between syndromic management and treatment based in realistic diagnostic options and local epidemiology are urgently needed.

Women commonly experience genital symptoms that are due to non-STIs like VCA or BV, which has been a challenge with syndromic diagnosis of STIs in women. In our model, testing negative for VCA or positive for BV on microscopic examination of vaginal swab wet preparations was associated with NG/CT infection. While microscopy is generally available in health centers, laboratory technicians may need to be (re) trained to diagnose TV, BV, or VCA on vaginal swab wet mount preparations. Microscopy could also potentially have a role in NG diagnosis**.** Gram-stained endocervical (or urethral) discharge, which is feasible in government health facilities, could be further explored. In our laboratory, 12/13 GeneXpert NG positive and 2/41 GeneXpert NG negative endocervical swabs from women were positive for NG on Gram stain. Of urethral discharge samples from men, 64/93 GeneXpert NG positive and 2/68 GeneXpert NG negative samples were positive for NG on Gram stain (unpublished data). The sensitivity of Gram stain is suboptimal and false positives may result when other Gram-negative coccobacilli including Moraxella osloensis, Moraxella phenylpyruvica, Kingella denitrificans, and Acinetobacter species are present. This is particularly true when microscopists have not been trained to distinguish intra from extra-cellular diplococci. That said, the cost is low and, with adequate quality control of microscopy, GeneXpert could, for example, be a backup for microscopy negative clients with an otherwise suggestive profile.

We did not include physical exam findings in our risk algorithm since physical exams are not routinely conducted in Rwandan government health facilities where trained clinicians and equipment for speculum exams may not be available. However, in our previous publication of data from this program (Wall et al., under review), we reported that distinguishing vaginal from endocervical discharges via physical exam may improve diagnostic performance. Visual examination of ulcer may also improve STI diagnosis [27]. We previously reported that most RPR+ patients did not report ulcers, which may be internal in women and difficult to see. Conversely, most men and women with ulcers were not RPR+. While physical exams are likely infeasible for all symptomatic patients in government clinic outpatient departments, genital exams for men are more feasible and targeted genital exams for women may be possible and useful.

We learned some relevant lessons when training the eight government healthcare providers who made diagnoses in the external validation cohort. Following didactic training, knowledge of the Rwandan National Criteria for syndromes substantially increased from 60 to 88% (*p* < 0.0001) and correct treatment for STI etiologies increased from 44 to 80% (p < 0.0001) (unpublished data). However, after providers were trained, overtreatment of NG/CT was more common using National Guidelines, with 59% of prescriptions being unnecessary. The cost-effectiveness of implementing an evidence-based risk algorithm along with improved provider trainings to identify and treat STI should be explored.

One important provider training topic is antibiotic resistance in NG. Initially, patients diagnosed with NG by GeneXpert at the CFHR site were treated with ciprofloxacin (first line using 2015 National Guidelines with ceftriaxone for cases of resistance) or ceftriaxone (first line in 2019 Guidelines) and asked to return in 2–3 weeks for re-testing. We found that NG positivity after treatment was significantly higher among those who received ciprofloxacin versus ceftriaxone (86% vs 15% measured 1–16 days after initial treatment). While some positive repeat tests may be due to persistence of nucleic acids detectable by GeneXpert for 2 weeks [28, 29] or re-infection, these results strongly suggest resistance to ciprofloxacin. As a result of our preliminary findings of resistance to ciprofloxacin, the 2019 National Guidelines changed the first line NG treatment to ceftriaxone (Wall et al., under review).

Another important training topic is partner notification to diagnose new cases and reduce reinfection in index cases. In the subset of the NG patients above, the proportion who were NG positive 17–30 days after Ceftriaxone treatment was 30% and this increased to 68% at > 30 days after Ceftriaxone treatment. Although without negative intervening tests we cannot be certain, the increase over time does suggest high rates of reinfection by an untreated partner. Partner notification involves identifying exposed sex partner(s) of index cases with STI, notifying them about their exposure, and offering testing, counselling and treatment [30]. Our program relied on patient referrals of sexual partners as described in National Guidelines. Other strategies include providing a referral slip for the index to give to partner(s) to seek treatment at a facility or asking the index case for contact information for their partners to allow clinic staff to communicate results directly by telephone or by mail [31]. A systematic review found that provider referral strategies may be more effective than patient referral in some populations [32].

Our program and data have some limitations. Data from an external validation cohort from another urban center would have improved the assessment of risk algorithm validity. Our findings are generalizable to symptomatic women who comprise most women seeking care at health facilities. However, many NG/CT cases are asymptomatic [33, 34], GeneXpert screening of high-risk populations may be warranted where feasible and affordable. Additionally, our findings are generalizable to urban women, who experience a larger proportion of STIs in Rwanda compared to rural women. We did not have the facilities to diagnose some etiologies of inflammation (e.g., M. genitalium) or ulcer (e.g., chancroid, lymphogranuloma venereum).

## Conclusions

Syndromic management guidelines in Rwanda can be improved with consideration of demographic, symptoms, and simple laboratory measures shown to be predictive of STI and non-STI dysbioses. Our data support use of a locally relevant, evidence-based risk algorithm to greatly reduce the number of untreated NG/CT cases in symptomatic Rwandan women. Our risk algorithm combined with provider training could be a cost-effective way to improve upon syndromic STI management and treatment in the Rwandan capital.

## Supplementary Information

**Additional file 1: Table S1.** Baseline characteristics and associations with CT or NG infection in symptomatic women, Kigali (*N* = 305): external validation cohort.

## Data Availability

The datasets analysed during the current study are available from the corresponding author on reasonable request.

## Abbreviations

AUC: Area under the receiver operating curve
BV: Bacterial vaginosis
CT: *Chlamydia trachomatis*
NPV: Negative predictive value
NG: *Neisseria gonorrhoeae*
PPV: Positive predictive value
RPR: Rapid plasma reagin
STI: Sexually transmitted infection
TV: *Trichomonas vaginalis*
VCA: Vaginal *Candida albicans*
WHO: World Health Organization
