# Supplementary material for: Developing and validating a risk algorithm to diagnose Neisseria gonorrhoeae and Chlamydia trachomatis in symptomatic Rwandan women
Source: BMC Infect Dis. 2021 Apr 28;21:392. doi: 10.1186/s12879-021-06073-z (PMC8080377; doi:10.1186/s12879-021-06073-z)
Supplement: Supplementary file 1 — Additional file 1: Table S1. Baseline characteristics and associations with CT or NG infection in symptomatic women, Kigali (N = 305): external validation cohort. [file 12879_2021_6073_MOESM1_ESM.docx]

| **Additional Table 1.** Baseline characteristics and associations with CT or NG infection in symptomatic women, Kigali (N=305): external validation cohort | | | | | | | |  |
| --- | --- | --- | --- | --- | --- | --- | --- | --- |
|  | **Total  (N=305)** | | **Either CT or NG (n=86)** | | **CT and NG Uninfected (n=219)** | | **p-value*** |  |
|  |  |  |  |  |  |  |  |  |
|  | **n/mean** | **Col%/SD** | **n/mean** | **Row%/SD** | **n/mean** | **Row%/SD** |  |  |
| **Demographics** | | | | | | | |  |
| **Age** |  |  |  |  |  |  |  |  |
| 25 or younger | 93 | 30% | 33 | 35% | 60 | 65% | 0.061 |  |
| Older than 25 | 212 | 70% | 53 | 25% | 159 | 75% |  |  |
| **Referrer** |  |  |  |  |  |  |  |  |
| Radio Advert | 94 | 31% | 24 | 26% | 70 | 74% | 0.490 |  |
| Other* | 211 | 69% | 62 | 29% | 149 | 71% |  |  |
| **Living and Marital Status Composite** |  |  |  |  |  |  |  |  |
| Married and Cohabiting | 148 | 49% | 43 | 29% | 105 | 71% | 0.747 |  |
| Other | 157 | 51% | 43 | 27% | 114 | 73% |  |  |
| **Education Level** |  |  |  |  |  |  |  |  |
| None/Primary | 174 | 57% | 44 | 25% | 130 | 75% | 0.193 |  |
| Secondary/Higher | 131 | 43% | 42 | 32% | 89 | 68% |  |  |
| **Employment Status** |  |  |  |  |  |  |  |  |
| Full-time employment | 118 | 39% | 32 | 27% | 86 | 73% | 0.740 |  |
| Part-time/Student/Jobless | 187 | 61% | 54 | 29% | 133 | 71% |  |  |
| **Sexual behaviors** | | | | | | | |  |
| **Number of partners in last 30 days** |  |  |  |  |  |  |  |  |
| None or one partner | 264 | 87% | 63 | 24% | 201 | 76% | <0.0001 |  |
| More than one partner | 41 | 13% | 23 | 56% | 18 | 44% |  |  |
| **Condom use during vaginal sex in the last three months** |  |  |  |  |  |  |  |  |
| Always (or did not have vaginal sex) | 17 | 6% | 1 | 6% | 16 | 94% | 0.085 |  |
| Sometimes | 93 | 30% | 30 | 32% | 63 | 68% |  |  |
| Never | 195 | 64% | 55 | 28% | 140 | 72% |  |  |
| **Number of days since sexual contact you suspect STI was acquired from** |  |  |  |  |  |  |  |  |
| 0-16 | 50 | 16% | 18 | 36% | 32 | 64% | 0.185 |  |
| >=17 | 254 | 84% | 68 | 27% | 186 | 73% |  |  |
| **Self-reported symptoms** |  |  |  |  |  |  |  |  |
| **Genital itching** |  |  |  |  |  |  |  |  |
| Yes | 198 | 65% | 51 | 26% | 147 | 74% | 0.180 |  |
| No | 106 | 35% | 35 | 33% | 71 | 67% |  |  |
| **Number of days with symptoms** |  |  |  |  |  |  |  |  |
| 1-10 | 73 | 19% | 25 | 34% | 48 | 66% | 0.188 |  |
| 11 or more | 232 | 60% | 61 | 26% | 171 | 74% |  |  |
| **HIV and Other STI Results** |  |  |  |  |  |  |  |  |
| **HIV Status** |  |  |  |  |  |  |  |  |
| Positive | 26 | 9% | 10 | 38% | 16 | 62% | 0.224 |  |
| Negative | 279 | 91% | 76 | 27% | 203 | 73% |  |  |
| **RPR Result** |  |  |  |  |  |  |  |  |
| Positive (1, 11 or greater) | 17 | 6% | 8 | 47% | 9 | 53% | 0.098 |  |
| Negative | 285 | 94% | 78 | 27% | 207 | 73% |  |  |
| **Candida** |  |  |  |  |  |  |  |  |
| Positive | 89 | 29% | 14 | 16% | 75 | 84% | 0.002 |  |
| Negative | 215 | 71% | 72 | 33% | 143 | 67% |  |  |
| **BV** |  |  |  |  |  |  |  |  |
| Positive | 122 | 40% | 44 | 36% | 78 | 64% | 0.013 |  |
| Negative | 183 | 60% | 42 | 23% | 141 | 77% |  |  |
| **Physical exam** |  |  |  |  |  |  |  |  |
| **Vaginal Inflammation or Discharge** |  |  |  |  |  |  |  |  |
| Yes | 225 | 74% | 68 | 30% | 157 | 70% | 0.187 |  |
| No | 80 | 26% | 18 | 23% | 62 | 78% |  |  |
| **Endocervical Inflammation or Discharge** |  |  |  |  |  |  |  |  |
| Yes | 110 | 36% | 57 | 52% | 53 | 48% | <0.0001 |  |
| No | 195 | 64% | 29 | 15% | 166 | 85% |  |  |
| *Heard from Friends/Walk-in/Pharmacy/Other/Invitation/Contact Partner/Internet | | | | | | | |  |
